# Supplementary figures and images for: Clemastine Induces an Impairment in Developmental Myelination
Source: Front Cell Dev Biol. 2022 Mar 17;10:841548. doi: 10.3389/fcell.2022.841548 (PMC8970281; doi:10.3389/fcell.2022.841548)

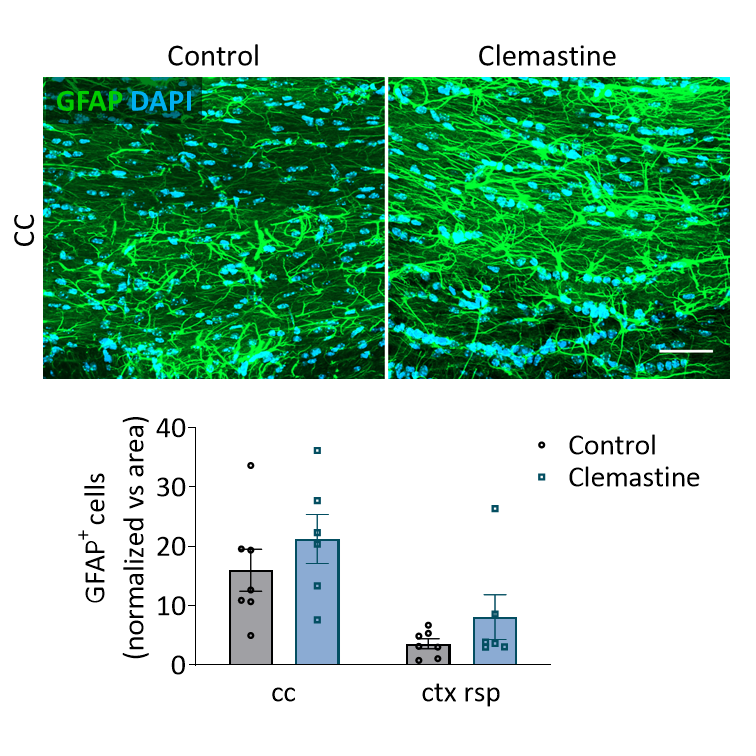

Supplement: Supplementary file 1 [file Image2.tif]

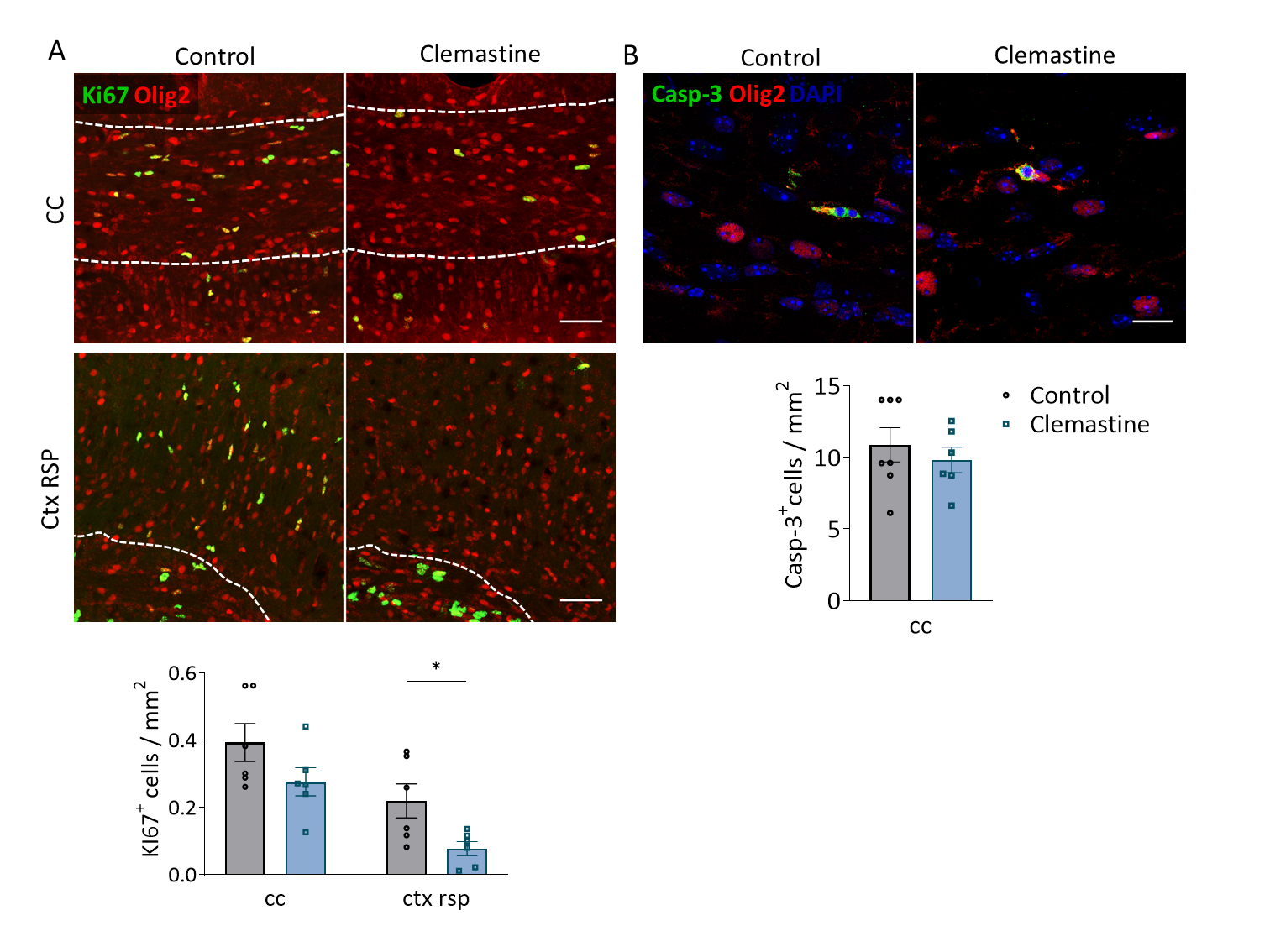

Supplement: Supplementary file 2 [file Image1.tif]
